# The traditional knowledge on stingless bees (Apidae: Meliponina) used by the Enawene-Nawe tribe in western Brazil

**DOI:** 10.1186/1746-4269-4-19

**Published:** 2008-09-15

**Authors:** Gilton Mendes dos Santos, Yasmine Antonini

**Affiliations:** 1 Departamento de Antropologia, Universidade Federal do Amazonas, UFAM. Av. Gen. Rodrigo Octávio Jordão Ramos, 3000 Campus Universitário. CEP 69077-000, Manaus, AM, Brazil; 2 Departamento de Ciências Biológicas, ICEB, UFOP. Campus Morro do Cruzeiro s/n. CEP: 34000-000, Ouro Preto, Minas Gerais, MG, Brazil

## Abstract

**Background:**

This paper presents the Enawene-Nawe Society's traditional knowledge about stingless bees. The Enawene-Nawe are an Aruak speaking people, indigenous to the Meridian Amazon. Specifically, they live in the Jurema River hydrological basin, located in the northwestern region of the Mato Grosso state.

**Methods:**

The stingless bees were sampled from two ecologically similar regions in the interior of Enawene-Nawe Land. The first sampling took place around the village, i.e., adjacent to houses, by the edge of the Iquê River, next to food leftovers, around human excrement, and simply when the insects were found flying or reposing on a human body. The second round of sampling happened from 29/10 to 02/11/94, during an expedition for honey collection that took place throughout the ciliar bushes of the Papagaio River, an important tributary of Juruena River. We sampled bees adjacent to their nests following the beehive inspection or during the honey extraction.

In this work, the main bee species of the sub tribe Meliponina, which were handled by the Enawene-Nawe, was identified, and a brief ethnographic description of the honey collection expeditions and its social-cosmologic meaning for the group was done.

**Results and Discussion:**

Similar to other indigenous people in Brazil, the Enawene-Nawe recognized 48 stingless bee species. They identified each bee species by name and specified each one's ecological niche. A brief ethnographic description of the honey collection expeditions and bees' social-cosmologic meaning for the group is included.

**Conclusion:**

We concluded that, as an example of other indigenous people, the Enawene-Nawe classify and identify the bees based not only on their structure and morphological aspects but also on the ecological, etiological, and social characteristics of the species.

## Background

The way that individuals perceive, identify, categorize and classify the natural world influences the way one thinks, acts, and expresses emotions in relation to animals and plants. The attitudes towards animals are characterized by our values, knowledge and perceptions as well as by the nature of the relationship that human beings have with particular animals [1].

Recently, Descola [2] redefined the old notion of *animism *and applied it to the context of amazonic indigenous theories, for which the animals and other natural beings are fully endowed with anthropocentric and social attributes.

In Greene's [3] conception, insects are seen as a representational category, once they have become metaphoric outcomes of other beings or their qualities. For example, the Mofu people, from Northeast Cameroon, Africa, generally project their own political and social behaviors onto the insects of their environment, especially ants and termites. There is an ant, known as "jaglavak" (*Dorylus *sp), that is considered the prince of the insects, and it is located on the top of the entomofauna hierarchy [4]. When a Mofu finds it, his behavior is characterized as respect and fear. Generally, he greets it by crackling his fingers, calling it *Bi *(boss) or *Bi erlam *(divinity) and bending over and touching his chest.

The studies that focus on the relationships between human societies and nature provide greater insight into anthropological knowledge. The second volume of the French anthropologist Claude Lévi-Strauss's, "From honey to ashes-mythological 2", is an exhaustive analysis of the South American indigenous mythology about the honey of the stingless bees. The author suggests that honey and tobacco, according to the Amerindian cosmologies, are antithetic and complementary elements (one is infra-cookery and the other is meta-cookery) in their representations of nature and culture.

Ethnoentomological studies, when analyzing the complex set of knowledge, thoughts, beliefs, feelings and uses of insects by the old and contemporary human communities, lead us to a deeper understanding about the way of life of the ethnic group being studied. In particular, such studies give us insight into human societal an ethnic group's interactions with the environment and their habits, traditions, and culture [5].

Records show that the ancient Mayans kept a tenuous relationship with the stingless bee. According to Cappa and Souza [6], bees were connected to religious questions and to cosmology in ancient Mayan culture. The importance of the stingless bees to Mayans is evident by the cultural adornments and statues that were made to represent the sacred bees and the bee god.

In Brazil, there are studies showing the importance of stingless bees for the cultural and economic life of the indigenous people there. For instance, the pioneering works of Darrel Posey describe the Kayapó's indigenous ethnoentomology [e.g., [7,8]]. His description and analysis show an intricate web of knowledge, which the Kayapó establish with the apicultural universe. Beyond a complex taxonomic system that they have devised, the Kayapó breed the stingless bees, widely using the honey in their daily and ritual life.

Like studies of the Kayapó, studies of the Guarani tribe (*Guarani-m' byá*), [9] and Pankararé tribe [10,11] showed that the bees are not seen separately from the ecosystem and that there are no forests without bees or vice versa. These studies showed the diverse knowledge that different indigenous cultures have about bees and wasps. They described more than 25 ethnospecies divided into two groups of insects. Furthermore, their knowledge included some of the following aspects: morphologic and ethological descriptions, distribution, nest building, seasonality, dispersion, practical aspects of handling and manipulation for the extraction of products, preservation and semi-domestication of species, and the use of their products [9].

Such indigenous knowledge of the stingless bees has helped to clarify the biology of some species [12]. The integration of indigenous knowledge is evident, as some species of stingless bees have popular and scientific names with indigenous origins. For instance, the term *Melipona mandaçaia *(*manda = guard, sai = pretty*): "*pretty guard*" describes a behavior found in that species such that there is always a guard at the entrance to the hive.

Although Brazil presents a wide diversity of indigenous people and approximately 450 known species of stingless bees, the studies about the role of these insects in the Amerindian culture are still incipient. It is necessary to have more accurate surveys about the knowledge that they have on the subject, which would certainly help the studies of conservation of the threatened species.

The Enawene Nawe are a small Amazonian tribe who live by fishing and gathering in Mato Grosso state, Brazil. They are a relatively isolated people who were first contacted in 1974. Today, they number over 500, living in large communal houses or *malocas*. These malocas radiate out from a central square where ritual and communal activities are performed.

The Enawene-Nawe are known for their adept fishing techniques. During the fishing season, the men build large dams across rivers and spend several months camped in the forest, catching and smoking the fish, which are then transported by canoe to their village. Fish is an essential part of their diet, and it plays a vital part in rituals such as Yãkwa, a four-month exchange of food between humans and spirits.

The Enawene-Nawe grow manioc and corn in gardens and gather forest products. Honey gathering is celebrated in *keteoko*, or the honey feast, when men collect large amounts of wild honey in the forest and hide it on their return to the village, only revealing it when the women start to dance. Unusual for an Amazonian tribe, they do not hunt or eat red meat.

In this work, we describe some of the dimensions of the Enawene-Nawe people's knowledge about the stingless bees.

## Methods

### Study area

**Figure 1:**
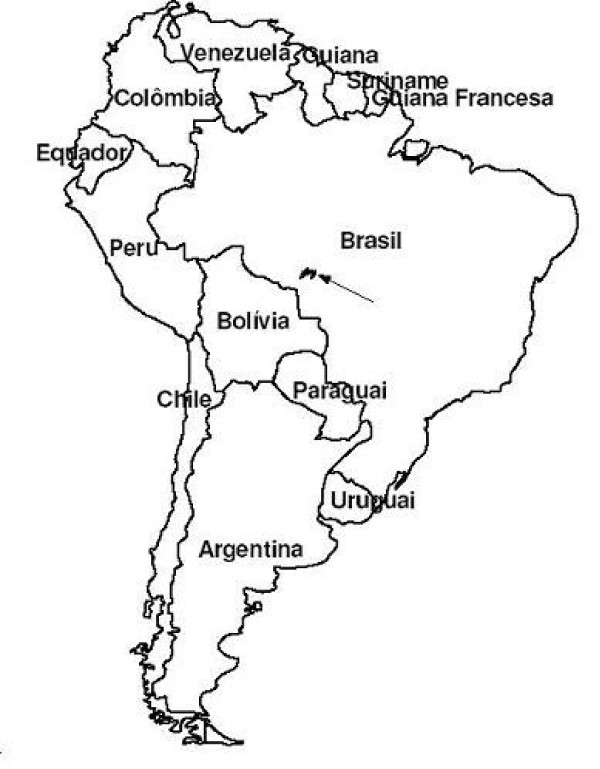
South America

The indigenous Enawene-Nawe land has an area of 7520 km^2 ^and is located at an average altitude of 300 m, between the latitudes 11°41' and 12°40' South and longitudes 59°55' and 58°24' West, with annual average rainfall of 2.000 mm and average temperature of 25°C (Figure 1).

Stingless bees were sampled in two ecologically similar yet distant regions in the interior of Enawene-Nawe land. The first round of samplings was conducted around the village, during four field visits: 17 to 23/09/93, 23 to 25/11/93, 24/02 to 06/03/94, and 18 to 23/07/94. Bees were captured in various areas around the village, i.e., near houses, or on at the Iquê River's edges, next to food leftovers, next to potable water, on human excrement, or simply when the insects were found flying or reposing on a human body.

The second round of sampling happened from 29/10 to 02/11/94, during an expedition for honey collection that took place throughout the ciliar bushes of the Papagaio River, an important tributary of Juruena River. We sampled bees adjacent to their nests following the beehive inspection or during the honey extraction.

Bees were captured with an insect net during the expeditions of honey and also the small incursions in the surroundings of the village with the help of local people [13]. After their capture, the bees were submitted (alive) to be identified by one of the Enawene, often men, young and old, in the village or in the honey encampment (*maha wesera*). Initially, the identification of different bee species was recorded in the native language. Then, the bees were taken to the Entomology Laboratory of the Agronomy and Veterinary Medicine Colleges of the Federal University of Mato Grosso for further traditional scientific identification.

## Results and discussion

The stingless bees and their products represent one of the main natural resources known to the Enawene-Nawe. Honey is one of the most appreciated natural products in Enawene-Nawe culture. Every flavorful thing that is tasted is compared to honey: "maha ikyari!" – "same as honey!" – they used to say. Questioned by a reporter during an indigenous game in Brazil about the source of the tribal men's strength, one Enawene immediately answered that it came from the honey. It is an important part of their diet. According to Ramos-Elorduy [14], stingless bee honey produces 4,53 kcal/kg, making it sufficiently caloric.

Collecting the honey occurs during the normal daily activities, at any time of the year, as well as during ritual periods. For rituals, the population divides itself into groups of four or more different encampments, living and exploring distinct regions of the territory for approximately 30 days. Only the men, both young and old, search for honey. The women stay at the encampment, cooking and taking care of the children. Cabeza de Vaca [15] also shows the importance of honey for the Guarani, for whom its meaning exceeds their nourishing necessities.

According to Rodrigues [9], the use of the products produced by stingless bees, such as honey, wax, cerume (a mix of wax and propolis), propolis, pollen and the bees themselves, was used by the Guarani m' byá for many purposes, i.e., as nourishment, traditional medicine, activities related to their spiritual and contemplative life and their hand-crafting. Beyond the nutritional value, the honey is also used for medicinal purposes by some tribes, such as the Guarani and Pankararé. For the Pankararé [10], the honey of many species is recommended for diabetes treatment, bronchitis, mycosis, throat aches and sexual impotence. Some of its other uses include use as a vermifugal and as an antidote against snakebites and rabid dog bites.

The Enawene-Nawe delineate different structures of the beehive. For instance, the entrance of the nest is called *iuxi*, the honey pots are called *enolone maha*, the honeycombs used for breeding are called *enesehi*, and the batume is called *edetera*, etc. The larvae, pre-pupae and pupae are indistinctly called *dioa *(child). However, the pupa (before emerging from the cell) is referred to as *enolone*, and the adult bees are referred to as *elalaykira*. According to Rodrigues [9], the Guarani m' byá people call the honeycombs *etãngue*, the honey pots *ei *and the pollen pots *evorakue*.

The Enawene-Nawe identify each bee species by associating its morphology to social and ecological characteristics, such as the place and structure of its nests, its behavior, the alimentary habits, the amount and flavor of the honey. In this work, we cited 48 different species (Table 1), all from the Meliponina sub-tribe. It was possible to cross-identify and describe 21 of them (Table 2).

**Table 1:** List of stingless bees named by Enawane-Nawe

*Koretosero*
*Kulata*
*Layrihi*
*Lokorihi*
*Loleylala*
*Lorese*
*Losewirara*
*Mayri*
*Natawane*
*Tahadodoya*
*Tahõhõ*
*Talaxawayri*
*Talokixita*
*Tariwase*
*Tawowene*
*Tayrikeytowa*
*Wareware*
*Wawixi*
*Waykywane*
*Wilalakowri*
*Woyawayoko*
*Yamayriri*
*Yawaloeta*
*Yolodoata*
*Yolotare *ou *Loreseri*

**Table 2:** Stingless bees species cited by the Enawene-Nawe with scientific identification.

Enawene-Nawe Name	Indigenous name	Scientific Name
*Irikayti*	tu	*Trigona dallatorreana*
*Koretosero*	mandassaia	*Melipona quinquefasciata*
	-	*Melipona schwarzi*
	-	*Melipona illustris*
	-	*Melipona seminigra abunensis*
*Kayalakase*	Borá	*Tetragona clavipes*
*Lorese*	Tubuna	*Scaptotrigona *sp., *Scaptotrigona bipunctata*
*Yolotare *ou *Loreseri*.	Jatahy	*Tetragonisca angustula*
*Tahadodoya*	Bora	*Tetragona goettei, Tetragona aff. dorsalis*
*Tahõhõ*	Dog bee	*Trigona branneri*
*Waykywane*	Mombuca	*Geotrigona mattogrossensis*
*Yamayriri*	Sanharó	*Trigona cilipes pellucida, Trigona truculenta*
*Kulata*	-	*Duckeola ghilianii*
	-	*Frieseomelitta trichocerata*
	-	*Scaura latitarsis*
	Miri	*Plebeia sp.*

## Ecological features of some stingless bee species

### Tetragona clavipes

**Enawene name **– *Kayalakase*.

**Indigenous name **– Bora.

The nest that we found was located in a tall tree hollow in the ciliar bush. The hive entrance was wide. It was made of the old nests' hardened propolis. It did not have a "trumpet" shape. It protruded relatively little. The pots were average sized, approximately 3 cm in height. There were developed involucres that had many cerume membrane layers.

In general, the honeycombs are in helical shapes. They have royal cells and propolis with medium viscosity. The batumen and caulking are made of cerume and propolis. The colonies are highly populated. They are aggressive, attacking by biting and flying around the eyes and ears. Another kind of aggressive behavior was noted by Salt [16], who observed that, in Colombia, these bees deposit propolis on the people who examine their nests. Dirty habits, like visiting feces, are not known in this species. According to Ducke [17], their honey is acidic. In Paraguay, Bertoni [18] wrote about this species: " [I]t's certainly the best honey producer, but it's always somewhat acidic". The Enawene-Nawe also consider the honey sour (*tiha*). For this reason, they throw away the old honey that remains in the colonies.

### Tetragona aff. dorsalis

**Enawene-Nawe name**: unknown

Indigenous name – Bora

We located the nest in a tree hollow found in the tall bush. The workers are not aggressive and the honey is good during the whole year.

### Tetragona goettei

Enawene Name – *Tahadodoya*.

Indigenous name – Bora

The nest was located in the trunk of a tree with a diameter of approximately 50 cm. The entrance was located about 30 cm from the ground in the ciliar bush. They are not aggressive, and the honey is considered very sweet, according to the Enawene-Nawe.

### Tetragonisca angustula

**Enawene-Nawe name **– *Yolotare *ou *Loreseri*.

**Indigenous name **– Jatahy.

We found nests in dead hollow trunks on the ground. The trunks had different diameters (from 30 to 50 cm). We found *T. angustula *at both sampling locations, around the village and around the honey collection encampment. The collectors often find them in forested areas, where the sunlight penetrates the nest entrance. The colonies are frequently well populated, and the number of bees from colony to colony varies from 2000 to 5000. The nests of *T. angustula *can have as many as 5000 breeding cells. Sometimes they are docile, but they can also be aggressive. When the bees display aggressive behaviors towards humans, they pinch the skin and roll up in the hair, but they soon calm down. "Dirty habits" are not known. Their honey is extremely flavorful, and it is produced in small amounts.

### Scaptotrigona bipunctata

**Enawene-Nawe name **– *Lorese.*

**Indigenous name **– Tubuna

We collected three species of the genus *Scaptotrigona*. Their nests had diameters of 40 to 50 cm, with the entrance situated approximately 7 m from the ground. The nest entrance was funnel-shaped and made of dark cerume. Their nests were found in the ciliar bush next to the Iquê River and the Papagaio River (adjacent to the honey collection encampment). The honey is known to be mildly acidic and very flavorful. When there is a lot of disturbance outside the hive, one can find many sentinels guarding the entrance.

The food pots have medium dimensions. They can range from 2.5 to 3 cm in height. The breeding honeycombs are generally helical, but sometimes they are horizontal. *Scaptotrigona *sp. build royal cells. The stored propolis has a medium viscosity. The batumen and the caulking are made with propolis and cerume. Generally, the colonies are very small. The size of the colonies varies from 2000 to 5000 bees. They are very aggressive bees, and they display the "tangle the hair" behavior. It's important to say that they are "dirty habit" bees because they visit excrement. In Guatemala, a tubuna subspecies frequently carries human excrement back to the nest to produce cerume. This species' honey is acidic and highly valued by the Enawene-Nawe.

### Trigona dallatorreana

**Enawene-Nawe name **– *Irikayti*

Indigenous name: Tu

### Trigona braueri

**Enawene-Nawe name **– *Irikayti*

Indigenous name: Abelha cachorro

According to the Enawene-Nawe, these two species of *Trigona *are considered to be one. Their hives usually hang from trees, connected to the tree solely by the upper portion of the nests. The bees are slightly aggressive. The little honey that they produce is highly valued by the Enawene-Nawe.

### Trigona cilipes pellucida

The nest was located in the ciliar bush. Honey production is small.

Indigenous name: Buhnide

### Trigona truculenta

**Enawene-Nawe name **– *Yamayriri*

Indigenous name: Sanharó

These bees are aggressive. They do not produce any honey.

### Melipona quinquefasciata

**Enawene-Nawe name **– *Koretosero*.

**Indigenous name **– Urusu

Most of the nest is found above ground, but the rest can reach more than 2 m deep in sand soil. They are very clean (they do not visit feces) and docile bees.

### Melipona seminigra abunensis

**Enawene-Nawe name **– *Koretosero*.

**Indigenous name **– Urusu

We found the nest in the hollow of a tree with diameter of approximately 1 m, and the entrance of the nest was located approximate 7 m high. It was located in a humid tall forest. They are not aggressive bees. According to the Enawene-Nawe, their honey is very sweet, and it is similar in flavor to the honey of the African bee (*tolelori*). The honey is clear and crystalline. The production of honey is good all year.

### Plebeia sp

**Enawene-Nawe name**: unknown

**Indigenous name **– Miri

Generally, the entrances are made of hardned propolis. It is generally short on the outside, pratically, not forming a tube, except in some cases. It is kept open during the night (with the exception of when there is danger of ant attacks). The breeding honeycombs are horizontal or helical. They build royal cells. The propolis is stored and very sticky and viscous. The batume and the caulking are made of cerume and propolis. In general, colonies are average in population. Sometimes the number of the beehive's inhabitants is very small. They are docile bees. They are known to lick the sweat of human skin, which is a possible vehicle for the transmission of mycosis. Their honey is not very flavorful.

### Duckeola ghilianii

**Enawene-Nawe name**: unknown

Indigenous name: Miri

We found the nest of this species at the honey collection encampment, by the Papagaio River, in hollow trees with diameters of approximately 40 cm. The entrances were approximately 7 m in height. The colonies ranged from having average to high populations. They are not aggressive, and their honey is not very sweet.

### Geotrigona mattogrossensis

**Enawene-Nawe name**: *waykywane*.

Indigenous name Mombuca

Their nest is subterranean, and their honey is sweet.

### The bees and the spiritual world

The Enawene-Nawe people say that the cosmos is made of many layers. Most of these layers are inhabited by different creatures, spirits, gigantic ogres and spectral beings. Out of all of these creatures, the most feared are the *jakayreti*, perverse spirits responsible for the disease and death of humans. The Enawene-Nawe dedicate important rituals to the *jakayreti*, marked by an extended socio-ecologic calendar, involving fishing and agricultural activities. There are also benevolent spirits, the *enore-nawe*, divine beings who inhabit a gigantic village, situated in the celestial platform of the cosmos. Considered to be Enawene-Nawe co-sanguineous relatives, these beings live in social harmony. They are physically good-looking and perfumed. They are the only beings who can expel the malefic presence of an *iakayreti*. The *enore-nawe *are the main *xamãs *(spirits of an all powerful god) and helpers in the healing sessions of sick people [19].

The Enewene-Nawe worship these celestial divinities in *salumã *and *kateokõ *rituals, marked by abundant offerings and consumption of honey, made possible by the long excursions of honey collection, from October to December. During these months, the whole population of the village lives in encampments that are spread about the indigenous territory in different micro-basins. In these encampments, the Enawene-Nawe collect large amounts of honey for ritualistic purposes. Stored in big calabashes or plastic receptacles, the honey is transported from the encampments to the village. There, it is consumed during the rituals.

The women organize the *kateokõ *ceremony. During *Kateokõ*, they present themselves publicly, singing and dancing, in the village's patio. This is a cue for one or more groups of men to leave and collect honey. After two or three days, the men suddenly return. Greased all over their bodies, they arrive, clamoring and spreading honey over houses and people alike. They chase the women, holding them firmly and smearing them with the apicultural product.

### Expedition for honey collection

According to the Enawene-Nawe, in the mythical past, all species of the stingless bees used to live in one giant tree, which was very thick and tall. In it, all bees made their nests. Some species used to live in the low part, and others lived in the upper section of the tree. From then on, they have spread themselves and started to live in the bushes. The *Apis *genus, later on known by them and generically denominated *tolelori*, were, according to their origin, donated by a celestial entity as one of his *xamãs*.

Never very far from a water source, the beehives are almost always located in the ciliar bushes. Thus, the bushes are the first places where the Enawene-Nawe searched for the bees. Once a nest is found and the bee type is identified, the next act is cutting down of the tree. For the honey extraction, it is necessary to open the trunks in transversal and lateral cuts. The structures that contain larvae and pupas are removed and dumped. The honeycombs are deposited into bowls or simply squeezed with the hands. The honey that is stored in the interior of the trunk is removed with the help of a sponge made of macerated palm leaves. When the collector identifies the queen bee, he normally rubs her against his eyes, believing that this gesture will bestow upon him the continued acumen to spot hives in future honey excursions. When the nest is completely destroyed, it is abandoned.

For the group expedition, the men divide themselves into groups of three or four. Soon after the sun rises, they leave, "tracking" through the woods. With baskets strapped to their backs, the honey collectors transport large, heavy knives, calabashes and pans.

As confirmed by our sampling results, most beehives are often found on the top of tall trees. Many others are found in rotten trunks of trees that have fallen.

The honey collector looks for older thicker trees to ensure the presence of bees. A touch with the back of the large knife on the trunk helps agitate the bees and draw them out, making it easier for their identification. Another good indicator that a hive is present is the presence of a tiny bird called a *xokwi*. It is an eager insect consumer, but its plume's color mixes with the forest green, making it hard to detect. However, a seasoned collector listens for its crow, which the Enawene-Nawe believe to proclaim, "It's here!" in reference to the presence of bees.

On an average collection day, the men find four or five stingless bee hives, which, on average, produce 2–3 kg of product for approximately five hours of work.

The honey collection generally happens while others are fishing in the rivers and streams next to the encampments. During honey collection, their diet is composed of honey, fish, fruits, and insects. The Enawene-Nawe usually add water to the honey, obtaining a solution (hydrohoney) that they call *mala*. They also use the honey to sweeten drinks and to eat with certain fruits, especially the palm *buriti *(*Mauritia flexuosa*).

The techniques for finding bee colonies differ among various indigenous people. According to Nogueira Neto [12], these various techniques used to locate beehives. Hollanda [20] cited seven authors who studied the nest-finding strategies performed by different indigenous societies. One strategy consists of capturing an worker and tying a bird plume to its body. The shadow cast allowed one to follow it throughout the bush. According to Rodrigues [9], the Guarani people locate colonies by their sense of smell. They claim that the smell surrounding the beehives is very peculiar. Other indigenous people use other ways to successfully determine the presence of bees, i.e., sunlight casting shadows of hives in the forest, landscape characteristics, the presence of certain vegetation, and the humidity [9].

Unlike other indigenous societies, such as the Guarani [9] and the Pankararé [11], the Enawene-Nawe do not breed bees.

### Observations of the enawene-nawe regarding the mutualism between the stingless bees and the harpy eagle (harpya harpija)

There is a group of tiny bees, known to the Enawene-Nawe as lorese (*Scaptotrigona sp*.), whose honey is highly valued. It is known to have a very peculiar habit: its attraction to the excrement and preys that remain on the gavião-real (*Harpia harpyia*), denominated by the Enawene-Nawe as *ayridini*. They claim that the lorese bees usually visit the beak and the nostril of this rapine bird and tranquilly nourish themselves with the food and other substances deposited there. Besides the head, the little lorese bees also visit the bird's body and even its cloaca, exploring the feces retained there. Often, the cloaca is visited during bouts of diarrhea caused by the consumption of monkey meat. The Enawene-Nawe also have observed that the monkey meat consumption provokes profuse sweating by the hawk, attracting those bees even more.

In a passage of the work "From honey to ashes" Levi-Strauss [21], calls attention to an observation made by the naturalist Henry Bates in one of his reports of his trip made to (insert place) in the 19^th ^century that the New World's stingless bees display "dirty habits". Bates suggests that stingless bees from the Amazon obtain less of their nutrition from flowers and more of it from tree sap and from bird excrement. Strauss also stressed the distinction between New World and Old World bees by citing Schwartz's observation that "the Meliponas are interested for the various matters, from the nectar and the pollen to the yield, the urine and the excrements" (apud Lévi-Strauss, op. cit.).

## Conclusion

Like other indigenous people, the Enawene-Nawe classify and identify bees based not only in their structure and morphologic aspects but also on the ecological, etiological, and social characteristics of the species. This work is the result of a preliminary survey of stingless bees and their cultural significance to the Enawene-Nawe people from the Meridian Amazon. As shown above, we could identify only 21 of the 48 species that the Enawene-Nawe cited, proving that bigger investments on field works should be made. The language barrier between our research group and the Enawene-Nawe people possibly contributed to our limited results.

The Enawene knowledge about the bees, connect the distinct dimensions of the cognitive competences ("of the human's spirit"), associating, concurrently, the bees to the social groups and to the nature beings. May be this is necessary to provide conditions for the ecologic and social-cultural equilibrium of the group.

## Competing interests

The authors declare that they have no competing interests.

## Authors' contributions

GMS carried out the field work, and GMS and YA wrote the paper. Both authors read and approved the final manuscript.

## References

[B1] Drews C (2002). Attitudes, knowledge and wild animals aspets in Costa Rica. Anthrozoös, Ashland.

[B2] Descola P, Kuper A (1992). Societies of nature and the nature of society. Conceptualizing society.

[B3] Greeene ES (1995). Ethnocategories, social intercourse, fear and redemption. Comment on Laurent. Society and Animals 3.

[B4] Seignobos C (1996). Les Mofu et leurs insectes. Trad Bota Appl.

[B5] Meyer-Rochow VB (1979). The diverse uses of insects in traditional societies. Ethnomedicine.

[B6] Cappas e Souza JP (1995). Os Maias e a Meliponicultura. Anales Asociacion Española de Entomologia Consejeria de Agricultura e Médio Ambiente, Cuencas.

[B7] Posey D (1979). Ethnoentomology of the Kayapó Indians of Central Brazil. PhD thesis.

[B8] Posey D (1983). O conhecimento entomológico Kayapó: etnometodologia e sistema cultural. Anuário.

[B9] Rodrigues AS (2006). Até quando o etnoconhecimento sobre as abelhas sem ferrão (Hymenoptera, Apidae, Meliponinae) será transmitido entre gerações pelos índios guarani m'byá da aldeia morro da saudade, localizada na cidade de São Paulo, estado de São Paulo, Brasil?. Sitientibus Série Ciências Biológicas.

[B10] Costa Neto EM (1999). A etnocategoria "inseto" e a hipótese da ambivalência entomoprojetiva. Acta Biológica Leopoldensia.

[B11] Costa-Neto EM (1998). Folk Taxonomy and cultural significance of "Abeia"(Insecta:Hymenoptera) to the Pankararé, Northeastern Bahia State, Brazil. J Ethnobiol.

[B12] Nogueira Neto P (1997). Vida e Criação de Abelhas Indígenas sem Ferrão.

[B13] Santos G Mendes dos (1995). Agricultura e coleta enawene-nawe: relações sociais e representações simbólicas. Estudo das potencialidades e do manejo dos recursos naturais na Área indígena Enawene-Nawe.

[B14] Ramos-Elorduy J (1990). Contenido calórico de algunos insectos comestibles de México. Revista da Sociedad Quimica del Mexico.

[B15] de Vaca Cabeza (1984). Naufrágios y comentários. Madri.

[B16] Salt G (1929). A contribution to the ethology of the Meliponinae. Trans Ent Soc London.

[B17] Ducke A (1916). Enumeração dos hymenopteros coligidos pela Comissão e revisão das espécies de abelhas do Brasil. Historia Natural, Zoologia.

[B18] Bertoni A, de W (1911). Contribuicion a la biologia de lãs avispas e abejas del Paraguay. Na Mus Nac Buenos Aires.

[B19] Santos G Mendes dos (2006). Da cultura à natureza – um estudo do cosmos e da ecologia dos Enawene-Nawe. PhD thesis.

[B20] Hollanda SB, Rio De Janeiro, José (1957). Caminhos e Fronteiras.

[B21] Lévi-Strauss C (2004). Do mel às cinzas (Mitológicas v 2.

